# *In vivo *induction of phase II detoxifying enzymes, glutathione transferase and quinone reductase by citrus triterpenoids

**DOI:** 10.1186/1472-6882-10-51

**Published:** 2010-09-17

**Authors:** Jose L Perez, Guddarangavvanahally K Jayaprakasha, Adriana Cadena, Elvia Martinez, Hassan Ahmad, Bhimanagouda S Patil

**Affiliations:** 1 Vegetable and Fruit Improvement Center, Department of Horticultural Sciences, Texas A&M University, College Station TX 77843-2119, USA; 2 Department of Chemistry, University of Texas Pan-American, Edinburg TX 78539, USA

## Abstract

**Background:**

Several cell culture and animal studies demonstrated that citrus bioactive compounds have protective effects against certain types of cancer. Among several classes of citrus bioactive compounds, limonoids were reported to prevent different types of cancer. Furthermore, the structures of citrus limonoids were reported to influence the activity of phase II detoxifying enzymes. The purpose of the study was to evaluate how variations in the structures of citrus limonoids (namely nomilin, deacetyl nomilin, and isoobacunoic acid) and a mixture of limonoids would influence phase II enzyme activity in excised tissues from a mouse model.

**Methods:**

In the current study, defatted sour orange seed powder was extracted with ethyl acetate and subjected to silica gel chromatography. The HPLC, NMR and mass spectra were used to elucidate the purity and structure of compounds. Female A/J mice were treated with three limonoids and a mixture in order to evaluate their effect on phase II enzymes in four different tissues. Assays for glutathione S-transferase and NAD(P)H: quinone reductase (QR) were used to evaluate induction of phase II enzymatic activity.

**Results:**

The highest induction of GST against 1-chloro-2,4-dinitrobenzene (CDNB) was observed in stomach (whole), 58% by nomilin, followed by 25% isoobacunoic acid and 19% deacetyl nomilin. Deacetyl nomilin in intestine (small) as well as liver significantly reduced GST activity against CDNB. Additionally isoobacunoic acid and the limonoid mixture in liver demonstrated a significant reduction of GST activity against CDNB. Nomilin significantly induced GST activity against 4-nitroquinoline 1-oxide (4NQO), intestine (280%) and stomach (75%) while deacetyl nomilin showed significant induction only in intestine (73%). Induction of GST activity was also observed in intestine (93%) and stomach (45%) treated with the limonoid mixture. Finally, a significant induction of NAD(P)H: quinone reductase (QR) activity was observed by the limonoid mixture in stomach (200%). In addition, the deacetyl nomilin treatment group displayed an increase in QR activity in liver (183%) and intestine (22%).

**Conclusion:**

The results of the present study suggests that, dietary intake of citrus limonoids may provide a protective effect against the onset of various cancers by inducing the activity of certain phase II detoxifying enzymes in specific organs.

## Background

According to recent studies, chemoprevention may be achieved by the use of exogenous factors to enhance endogenous mechanisms that reduce the risk of cancer development due to exposure to different environmental factors. Some of these exogenous factors are immunizations, drugs, supplements and dietary constituents. Several studies have attributed the biochemical composition of various fruits and vegetables as instrumental in reducing the risk of cancer development [1,2]. One significant mechanism by which this is accomplished is by the induction of the activity of phase II detoxifying enzymes by bioactive components of fruits and vegetables [3,4]. Among these detoxifying enzymes are glutathione S-transferase (GST) and NAD(P)H: quinone reductase (QR). GST isoenzymes are thought to play a crucial physiological role in the initial stages of detoxification of various xenobiotics including alkylating agents [5]. Generally, an increase in the activity of GST enhances the ability of an organism to detoxify numerous potentially harmful xenobiotics. It has been reported that substances that increases the activity of GST can be potential chemopreventive agents with the ability to inhibit chemically induced cancer formation [6]. Similarly, QR protects against cytotoxicity by catalyzing a two electron reduction of quinones that arise from one electron reduction. Therefore, an increase in the levels QR is positively correlated with chemoprevention as well [7].

**Figure 1:**
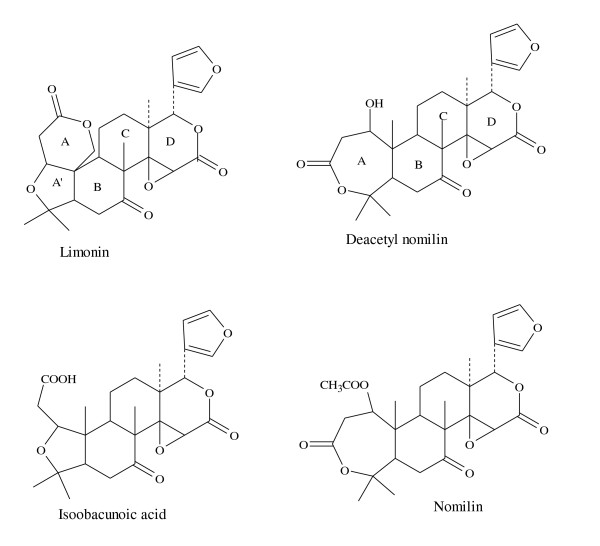
**Structures of limonoids purified from sour orange and used for the animal experiment**.

Citrus fruits contain many classes of bioactive compounds that may have the potential to reduce the risk of cancer [8]. From these, a group of chemically related triterpene derivatives called citrus limonoids are of particular interest (Figure 1). Many studies, using limonoids, provided evidence of their protective effects in cancer prevention, cardiovascular, viral, and other chronic conditions [9-13]. Cell culture studies from our lab resulted in a promising outcome while evaluating the effect of limonoids on human cancer cell lines [14-17]. It has been suggested that one mechanism by which citrus limonoids are chemopreventive is that the structure of specific limonoids induce the activity of phase II enzymes therefore conjugating xenobiotics to produce a hydrophilic substance that is more readily excreted in urine [18]. Initial animal studies have begun to evaluate how limonoids differing in structure may alter phase II enzymatic activity [9,19-21]. The data presented in this study further enhances and reinforces current knowledge on the possible chemopreventive properties of citrus limonoids. In addition, due to the variability in GST isoenzymes present in various tissues, 4NQO and CDNB substrates were used in this study to obtain a more comprehensive picture of the influence of citrus limonoids on GST activity [9].

The main purpose of this study was to elucidate the function of various structurally unrelated citrus limonoids in the induction of phase II enzymes such as GST and QR. In order to understand structure-activity relationship of limonoids on biological activity, we analyzed the induction potential of specific citrus limonoids (Figure 1) with a closed A ring (nomilin, deacetyl nomilin) and open A ring (isoobacunoic acid). Furthermore, limonoid mixture (limonin, nomilin and isoobacunoic acid) was evaluated for the potential synergistic induction capabilities. The relationship between the various structural properties of citrus limonoids and induction of phase II detoxifying enzymes is vital for understanding the cancer prevention properties of various agents.

## Methods

### Materials

Silica gel and solvents were purchased from Fisher scientific (Pittsburgh, PA). The following chemicals were purchased from Sigma Aldrich (St. Louis, MO, USA): β-NADPH reduced tetrasodium salt, 2,6-dichlorophenol-indophenol (DCPIP), 1-chloro-2,4-dinitrobenzene (CDNB), 4-nitroquinoline 1-oxide (4NQO), glutathione reduced FAD disodium salt. Bovine Serum Albumin was obtained from Intergen (Purchase, NY).

### Purification of limonoids

Fruits of *Citrus aurantium L. *were harvested during October-November from Citrus Center Orchard, Texas A&M University-Kingsville, TX. Seeds were separated, air dried at 25°C and powdered using a blender. Seed powder (2.2 Kg) was defatted with hexane (4 L) and extracted by Soxhlet with ethyl acetate (4 L) for 8 h. The extract was filtered and concentrated under vacuum. This material was infused with silica gel and loaded to silica gel column chromatography. The column was eluted with different concentrations of chloroform and acetone to obtain three potential putative compounds. Compound (1), (2) and (3) were eluted with chloroform, chloroform: acetone (9:1) and (5:5) respectively. The mixture was obtained in between these fractions.

### HPLC analysis

The purity of the isolated compounds were analyzed by HPLC (Perkin Elmer Series pump 2000, Boston, USA) fitted with C_18 _Phenomenex Gemini series column (Torrance, CA, USA), 5 μm particle size, (250 × 4.6 mm) and coupled with a Perkin Elmer Series 2000 Autosampler and a Perkin Elmer Diode Array detector 235C. The gradient mobile phase consisted of 0.03 mM phosphoric acid (A) and acetonitrile (B) with the flow rate of 1.0 ml/min. The elution program involved a linear gradient from 5 to 50% B in A for 0-38 min and 5% B in A for 35-43 min followed by 5 min of equilibrium with 5% B. The eluted compounds were detected by their absorbance at 210 nm. The purity of the compounds and mixture composition was analyzed according to our published methods [6,22]. Further structures of the isolated compounds were confirmed by spectroscopic methods and results were compared to our published papers [7-9,22]

### Animal studies

Female A/JOlaHsd 8-9 weeks old mice were purchased from Harlan Sprague-Dawley Laboratory (Indianapolis, IN). Procedures, recommended by the federal agencies and approved by the University of Texas-Pan American Institutional Animal Care and Use committee were followed for animal handling, treatment, and euthanasia. The mice were fed AIN-76 semi-purified custom diet without vitamin E (MPBiomedical, Solon, OH) and tap water *ad libitum*. The mice were kept in plastic cages in an environmentally controlled room on a 12 h light/12 h dark cycle.

The mice were divided into four experimental groups and one control group. Each group consisting of four mice (n = 4). The experimental groups were treated with nomilin, deacetyl nomilin, isoobacunoic acid and a limonoid mixture consisting of limonin, nomilin, and isoobacunoic acid. Each treatment (20 mg) was suspended in DMSO: corn oil (1:1) (v/v) and administered by oral gavage once every two days. The control group was given the corresponding DMSO: corn oil (1:1) treatment. The total treatment was composed of four administrations for each of the compounds being investigated. Forty-eight hours after the last treatment the mice were sacrificed by cervical dislocation. Lung, small intestine, whole stomach, and liver were harvested immediately after sacrifice and washed with cold PBS. Excised tissues were weighed and homogenized using a Pro200 homogenizer with PBS (10 mM), pH 7.0, containing β-mercaptoethanol (1.4 mM) to obtain a 10% (w/v) homogenate. The cell extracts were centrifuged at 22,000 × g for 45 min in a refrigerated Beckman Avanti 30 centrifuge. Following centrifugation, the supernatant was carefully removed and stored at -20°C until further use.

### Determination of enzyme activities and protein

GST activity was determined using CDNB by the modified method from previous publications [23,24]. This system involves the addition of GSH to CDNB, a nucleophilic aromatic substitution that occurs via an addition-elimination sequence involving a short lived σ-complex intermediate [25]. In addition, GST activity determination using 4NQO was also evaluated in order to explore induction of different GST isoenzymes in different organs. This assay is modeled after the method developed by Stanley et al [26]. Furthermore, an assay for the examination of QR activity was employed for evaluating the potential of limonoids to induce activity. The QR assay was adapted from the method reported by Wang et al. [27]. Detail for all assays performed can be referred to in our previous publications [9].

All enzyme assays were performed using a spectrophotometer equipped with enzyme kinetic software and programmed to calculate enzyme units. The amount of enzyme that used 1 μmole of substrate per minute at 25°C is equivalent to one unit of enzyme activity. The protein contents of the samples were quantified by Bradford's method [28]. The absorbance was read at 595 nm. Bovine serum albumin was used as a standard. Each organ homogenate represented one sample from individual mice; all assays were performed in triplicates. Student's t-test was used to assess the significance of the data. P-values of p < 0.05 were considered significant.

## Results

**Figure 2:**
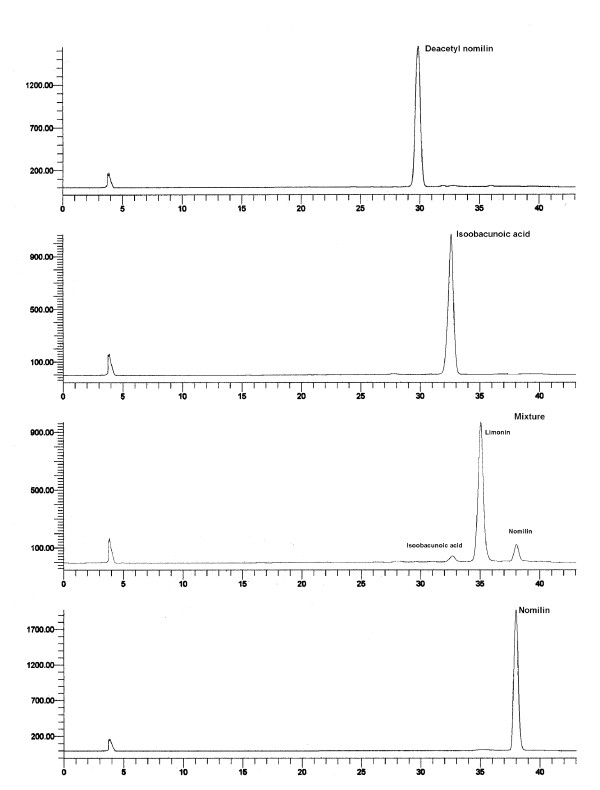
**HPLC profile of limonoids and a mixture obtained from sour oranges**.

Purification of ethyl acetate extract of *Citrus aurantium *yielded three compounds (1, 2, and 3) and a mixture. The purity of the isolated compounds and composition of the mixture were analyzed by HPLC as per our previous method [22] and the chromatograms have been presented in Figure 2. The mixture contains limonin (87.1%), nomilin (7.1%) and isoobacunoic acid (3.8%). Further, structures of the pure compounds (1, 2 and 3) were identified as nomilin, isoobacunoic acid and deacetyl nomilin respectively by NMR studies and chemical shifts were compared with our published data [7-9,29].

The biological effect of three limonoids and the mixture of limonoids were investigated on four organs of female A/J mice on the induction of GST activity (Table 1). Among the limonoids tested, the highest induction of GST was observed against CDNB by nomilin (58%), followed by isoobacunoic acid (25%) and deacetyl nomilin (19%) in stomach. Interestingly, deacetlylnomilin in intestine as well as in liver demonstrated significant reduction of GST activity. Furthermore the mixture of limonoids and isoobacunoic acid in liver also demonstrated significant reduction of GST against CDNB. No significant changes were observed in any of the treatment groups in lung homogenates.

**Table 1:** GST Specific activity against 1-chloro-2,4-dinitrobenzene, CDNB (Specific activity expressed as units/mg protein)

Samples	Stomach	Intestine	Liver	Lung
Control	0.728 ± 0.05	1.260 ± 0.13	2.520 ± 0.11	0.399 ± 0.07
Mixture	0.757 ± 0.02	1.191 ± 0.11	1.833 ± 0.26*	0.414 ± 0.02
Nomilin	1.152 ± 0.16**	1.122 ± 0.24	2.223 ± 0.39	0.395 ± 0.01
Deacetyl nomilin	0.872 ± 0.09**	0.715 ± 0.18*	1.582 ± 0.24*	0.449 ± 0.01
Isoobacunoic Acid	0.914 ± 0.13**	0.825 ± 0.84	1.390 ± 0.02*	0.365 ± 0.01

Results are means ± SD (n = 4)

* indicates statistically significant (p < 0.05) reduction of activity.

** indicates statistically significant (p < 0.01) induction of activity.

GST activity was measured against 4NQO, in four organs mentioned earlier and the results are presented in Table 2. The most noticeable induction of GST activity against 4NQO was observed in intestine homogenates compared to control. Nomilin showed significant induction of GST in intestine (280%), followed by the limonoid mixture (93%) and deacetyl nomilin (73%) also induced GST activity in intestine homogenates. In stomach homogenates, nomilin and the limonoid mixture significantly (p < 0.05) induced GST activity, 75% and 45% respectively, against 4NQO. In the other organs, lung and liver, no significant changes were observed.

**Table 2:** GST Specific Activity against 4-Nitroquinoline 1-oxide (Specific activity expressed as units/mg of protein)

Samples	Stomach	Intestine	Liver	Lung
Control	0.519 ± 0.06	0.322 ± 0.06	1.720 ± 0.34	0.414 ± 0.19
Mixture	0.755 ± 0.04**	0.624 ± 0.18**	1.765 ± 0.40	0.426 ± 0.04
Nomilin	0.927 ± 0.12**	0.905 ± 0.13**	2.045 ± 0.25	0.351 ± 0.08
Deacetyl nomilin	0.594 ± 0.12	0.558 ± 0.17 **	1.551 ± 0.30	0.431 ± 0.04
Isoobacunoic Acid	0.592 ± 0.02	0.604 ± 0.57	1.179 ± 0.05	0.374 ± 0.02

Results are means ± SD (n = 4)

* indicate statistically significant (p < 0.05) reduction of QR activity was observed.

** indicate statistically significant (p < 0.01) induction of QR activity was observed.

Table 3 depicts quinone reductase (QR) activity in liver, intestine, lung and stomach homogenates. In stomach homogenates, significant induction of QR activity was observed using limonoid mixture (200%) treatment group. Deacetyl nomilin also showed QR induction in liver (180%) and intestine (22%) homogenates. Similar to GST, none of the limonoids significantly induced QR activity in lung homogenates. Isoobacunoic acid, however, showed significant reduction of QR activity compared to the control in stomach.

**Table 3:** Quinone Reductase Specific Activity (Specific activity expressed as units/mg of protein)

Samples	Stomach	Intestine	Liver	Lung
Control	6.592 ± 0.60	3.838 ± 0.54	0.618 ± 0.06	0.377 ± 0.12
Mixture	13.372 ± 4.17**	3.544 ± 0.92	0.623 ± 0.10	0.374 ± 0.05
Nomilin	6.226 ± 0. 71	2.892 ± 0.43	0.662 ± 0.17	0.354 ± 0.02
Deacetyl nomilin	6.113 ± 2.12	4.702 ± 0.43**	1.137 ± 0.36**	0.356 ± 0.04
Isoobacunoic Acid	4.982 ± 1.19*	3.229 ± 2.57	1.477 ± 0.78	0.349 ± 0.03

Results are means ± SD (n = 4)

* indicate statistically significant (p < 0.05) reduction of QR activity was observed

** indicate statistically significant (p < 0.01) induction of QR activity was observed.

## Discussion

Recent research on citrus limonoids has specifically focused on isolation, elucidation of structures, and the effects of these compounds on different biological systems [9-16,29-32]. One focal point in citrus limonoid research has been on their ability to induce the activity of phase II detoxification enzymes. A recent study by Wark *et al*. suggests that habitual consumption of fruits and vegetables positively correlated with human rectal GST activity [33]. It is thus possible that an increase in the activity of phase II enzymes enhances the ability of an organism to effectively detoxify carcinogens. Therefore, any substance that increases the activity of phase II enzymes may be a potential chemopreventive agent with the ability to inhibit chemically induced cancer formation [21]. Several studies have attributed the induction of these enzymes to the structural components of limonoids. The furan moiety has been thought to be one of the components responsible for induction of GST activity [21]. Lam et al. [34] studied two furan containing diterpenes, kahweol and cafestol, which were shown to induce an increase in the activity GST in various tissues in mice. In a later study, eight citrus limonoids were tested for induction of GST activity in mice and the structural features of different citrus limonoids and their effect on GST activity was reported [21]. Other structural features that seem to play a role in the induction of GST activity are the A and A' rings, and modification to the B ring in limonoids [9,21].

Quinone reductase activity has also been observed along with other phase II enzymes [35]. QR protects against cytotoxicity, and an increase in its levels is positively correlated with chemoprevention. QR levels increased along with the levels of other chemopreventive enzymes which were induced by chemicals with diverse functional groups [35]. QR catalyses the two electron reduction of quinones to protect cells from free radicals and reactive oxygen species that arise from one electron reduction [36]. In a previous study, nimbolide, a limonoid isolated from neem flowers, showed induction of QR activity in Hepa1c1c7 cells [37].

In our study, nomilin, which is composed of intact A, B, C, and D rings showed the highest induction activity in the GST assays. Results from this study and previous studies consistently exhibited nomilin as an inducer of GST [19,21] It is possible that, nomilin possesses most of the structural features that are reported to be essential in the induction of GST activity. Deacetyl nomilin has a structural similarity to nomilin with the exception of the deacetylation of the A ring (Figure 1). Deacetyl nomilin induced GST in some organs, while also inducing QR activity in liver and intestine. These results reinforces our observation that the composition of the A ring seems to be critical for the induction of phase II enzymes.

Isoobacunoic acid has an intact A' ring and an open A ring, while the rest of the limonoid structure is similar to nomilin. Isoobacunoic acid induced GST activity against CDNB was only observed in stomach homogenates. It is interesting that induction of GST activity was observed even in the absence of an intact A ring (Table 1). It is therefore possible that other structural components of limonoids structure may influence enzyme induction.

The limonoid mixture used in this study induced GST activity against 4NQO in stomach and liver homogenates and induction of QR activity was observed in stomach homogenates (Table 3). The limonoid mixture was composed of limonin (87.1%), nomilin (7.1%), and isoobacunoic acid (3.8%). In previous studies [9,21], it was concluded that limonin was ineffective as a GST inducer. This was attributed to the fact that the structure of limonin is composed of intact A and A' rings. In the current study, the ability of the limonoid mixture to induce GST activity was probably due to the presence of nomilin in the mixture. It is likely that minor components of the mixture possibly have some biological attributes.

In this study, none of the limonoids or mixture had effect on GST or QR activity in the lung. This phenomenon could be due to the fact that the composition of GST isoenzymes varies in mammalian tissues. Previous reports suggest that variability of GST isoenzymes is influenced by tissue, species, and gender [38-40]. Most major mammalian GST isoenzymes are grouped into four major classes namely, α, μ, π and θ [41]. Accumulating evidences suggest that certain citrus limonoids can induce detoxifying enzymes, mainly GST [9-12,19-21]. In majority of these studies, nomilin seems to be the strongest inducer of GST activity against CDNB. During evaluation of induction of GST activity, CDNB was used as a general substrate, which did not reveal any substrate preference for any of the three major GST isozymes α, μ, π [42]. A later study showed that the specific activity of α, μ, π isozymes varied widely when CDNB was used as a substrate [43]. One study suggests that the GST μ isoenzyme is more efficient in the conjugation of 4-nitroquinoline 1-oxide (4NQO) [44]. Furthermore, it was also demonstrated that one of the GST isoenzymes in stomach is efficient in the conjugation of glutathione but was not detected in lung homogenate from A/J female mouse [39]. Other studies suggested that the gender of the organism may also influence isoenzyme composition along with GST differences among tissues [39]. It is therefore possible that the lack of induction of GST activity in lung by citrus limonoids may be due to the fact that the induced GST isoenzymes, if any, were not specific for CDNB conjugation.

In addition to the inactivity of limonoid in lung homogenates, certain limonoids showed reduction of GST activity in different organs. Previous studies have shown that GST activity can be inhibited, reversibly and irreversibly by various compounds [45]. It has been proposed that possible inhibition mechanisms are the covalent bonding of compounds to the GST enzyme, competitive inhibition against CDNB, and noncompetitive inhibition against GSH [46-48]. The inhibition of GST could prevent the possible inactivation of chemotherapeutic drugs and thus could be beneficial for chemotherapeutic reason [49].

## Conclusion

In this study, the citrus limonoids have shown specific induction of GST and QR with one or more substrates. The induction of these enzymes illustrates the potential chemopreventive role of citrus limonoids. It is possible that, an adequate intake of citrus fruit may detoxify phase II enzymes in the body, which may have potential benefits in prevention of cancer and other xenobiotic related diseases. Further studies are needed to determine how these limonoids might be reducing phase II enzyme activity.

## Competing interests

The authors declare that they have no competing interests.

## Authors' contributions

JLP carried out assays, sample collection and preparation, and drafted the manuscript. GKJP isolated, and identified limonoids, edited the manuscript and coordinated the study. AC and EM prepared samples and carried out assays. HA and BSP coordinated study, edited the manuscript and provided grant for this study.

## Pre-publication history

The pre-publication history for this paper can be accessed here:

http://www.biomedcentral.com/1472-6882/10/51/prepub
